# Supplementary material for: Content-rich biological network constructed by mining PubMed abstracts
Source: BMC Bioinformatics. 2004 Oct 8;5:147. doi: 10.1186/1471-2105-5-147 (PMC528731; doi:10.1186/1471-2105-5-147)
Supplement: Additional File 2 — The original results of the above study (non-essential files are deleted to keep the file size under the limit set by BMC bioinformatics). [file 1471-2105-5-147-S2.bz2 › chilibotAdditionalFile2/dip50/175ID7592797E594/html/IFNAR1_IFNA2.html]

 


 **IFNAR1** and **IFNA2** 
  
Found 467 abstracts in PubMed, retrieved 50.  
 

 What does Google say? 
 PDF only 
| .edu only 

---

- Clin Exp Immunol, 2003   **Anti cytokine autoantibodies in autoimmunity preponderance of neutralizing autoantibodies against interferon alpha **[IFNAR1]**, interferon omega and interleukin 12 in patients with thymoma and or myasthenia gravis.** .
  We have screened for spontaneous anticytokine autoantibodies in patients with infections, neoplasms and autoimmune diseases, because of their increasingly reported co occurrence.
  We tested for both binding and neutralizing autoantibodies to a range of human cytokines, including interleukin 1alpha IL 1alpha , IL 1beta, IL 2, IL 4, IL 6, IL 8, IL 10, IL 12, IL 18, interferon alpha2 **[IFNA2]** IFN alpha2 , IFN omega, IFN beta, IFN gamma, tumour necrosis factor alpha TNF alpha , transforming growth factor beta 1 TGF beta1 and granulocyte macrophage colony stimulating factor GM CSF , in plasmas or sera.
  With two notable exceptions described below, we found only occasional, mostly low titre, non neutralizing antibodies, mainly to GM CSF.
  also to IL 10 in pemphigoid.
  Strikingly high titre, mainly IgG, autoantibodies to IFN alpha2, IFN omega and IL 12 were common at diagnosis in patients with late onset myasthenia gravis LOMG , thymoma T but no MG TMG and especially with both thymoma and MG together TMG .
  The antibodies recognized other closely related type I IFN alpha subtypes, but rarely the distantly related type I IFN beta, and never detectably the unrelated type II IFN gamma.
  Antibodies to IL 12 showed a similar distribution to those against IFN alpha2, although prevalences were slightly lower.
  correlations between individual titres against each were so modest that they appear to be entirely different specificities.
  Neither showed any obvious correlations with clinical parameters including thymoma histology and HLA type, but they did increase sharply if the tumours recurred.
  These antibodies neutralized their respective cytokine in bioassays in vitro.
  although they persisted for years severe infections were surprisingly uncommon, despite the immunosuppressive therapy also used in most cases.
  These findings must hold valuable clues to autoimmunizing mechanisms in paraneoplastic autoimmunity.

  - J Viral Hepat, 2001   **Expression of interferon alpha **[IFNAR1]** subtypes in peripheral mononuclear cells from patients with chronic hepatitis C a role for interferon alpha5.** .
    Interferon IFN alpha is a family of antiviral proteins encoded by different genes.
    The biological significance of the existence of various IFN alpha subtypes is not clear.
    We have investigated the interferon system in chronic hepatitis C virus HCV infection, a disease that responds to interferon alpha2 **[IFNA2]** therapy in only a limited proportion of cases.
    We analysed the expression of interferon regulatory factor IRF 1, IRF 2, and IFN alpha subtypes in nonstimulated and Sendai virus stimulated peripheral blood mononuclear cells PBMC from HCV infected patients and healthy controls.
    We observed that the IRF 1 mRNA and IRF 1 IRF 2 ratios were increased in PBMC from hepatitis C patients with respect to normal subjects.
    Sendai virus stimulation of PBMC led to a significant increase in the levels of IRF 1, IRF 2 and IFN alpha mRNAs and in the production of IFN alpha protein with respect to basal values in healthy controls as well as in patients with HCV infection.
    In addition, we found that while natural HCV infection induced increased IFN alpha5 expression in PBMC, in vitro infection of these cells with Sendai virus caused a raise in the expression of IFN alpha8 in both patients and normal controls.
    In summary, our results indicate that virus induced activation of the IFN system in human PBMC is associated with selective expression of individual IFN alpha subtypes, IFN alpha5 being the specific subtype induced in PBMC from patients with chronic HCV infection.

    - Proc Natl Acad Sci U S A, 2001   **Prolonging the half life of human interferon alpha **[IFNAR1]** 2 in circulation Design, preparation, and analysis of 2 sulfo 9 fluorenylmethoxycarbonyl 7 interferon alpha **[IFNAR1]** 2.** .
      Polypeptide drugs are generally short lived species in circulation.
      In this study, we have covalently linked seven moieties of 2 sulfo 9 fluorenylmethoxycarbonyl FMS to the amino groups of human interferon alpha2 **[IFNA2]**.
      The derivative thus obtained FMS 7 IFN alpha2 has approximately 4% the biological potency and 33 4% the receptor binding capacity of the native cytokine.
      Upon incubation, FMS 7 IFN alpha2 undergoes time dependent spontaneous hydrolysis, generating active interferon with t 1 2 values of 24 2 h at pH 8.5 and 98 10 h at pH 7.4.
      When native IFN alpha2 is intravenously administered to mice, circulating antiviral activity is maintained for a short duration and then declines with t 1 2 = 4 0.5 h, reaching undetectable values at approximately 18 h after administration.
      With intravenously administered FMS 7 IFN alpha2, there is a lag period of 2 h, followed by a progressive elevation in circulating antiviral active protein, which peaked at 20 h and declined with t 1 2 = 35 4 h.
      FMS 7 IFN alpha2 is resistant to alpha chymotrypsin digest and to proteolytic inactivation by human serum proteases in vitro.
      We have thus introduced here an inactive IFN alpha2 derivative, which is resistant to in situ inactivation and has the capability of slowly reverting to the native active protein at physiological conditions in vivo and in vitro.
      Having these attributes, FMS 7 IFN alpha2 maintains prolonged circulating antiviral activity in mice, exceeding 7 8 times the activity of intravenously administered native cytokine.

      - J Surg Oncol, 2000   **Prospective phase II trial of alternating intravesical Bacillus Calmette Guérin BCG and interferon alpha **[IFNAR1]** IIB in the treatment and prevention of superficial transitional cell carcinoma of the urinary bladder preliminary results.** .
        BACKGROUND AND OBJECTIVE Evaluate the efficacy and toxicity of alternating intravesical instillation of Bacillus Calmette Guerin BCG and Interferon alpha2 **[IFNA2]** b IFN in the treatment and prevention of recurrence of superficial transitional cell carcinoma TCC of the urinary bladder.
        METHODS Patients with Ta, T1 tumors and carcinoma in situ, either recurrent TaG1, T1G1 or primary recurrent TaG2 TaG3, T1G2, T1G3 and Tis T Tumor stage, G grade are eligible.
        All patients received intravesical BCG 81 mg on Weeks , 5 and 7 and IFN 100 million units on Weeks , 6 and 8.
        Cystoscopy performed 4 weeks after completion of therapy, and every 3 months thereafter.
        RESULTS There was a total of 37 patients.
        Thirteen had TaG 3 T1G T1G TaG TaG T1G3 and 7 Tis 5 concurrent with other above tumors .
        Index lesion cleared in 7 10 patients.
        With a median follow up of 26.2 month, 22 patients 59% failed above therapy.
        Median time to treatment failure was 7 months.
        Seven, 6 and 9 patients recurred at a higher, lower and same stage or grade respectively.
        No grade 3 or 4 toxicity was encountered.
        CONCLUSIONS Alternating intravesical BCG and IFN is effective and well tolerated therapy for superficial TCC of urinary bladder.

        - Cell Death Differ, 1999   **Interferon alpha **[IFNAR1]** induces apoptosis in human KB cells through a stress dependent mitogen activated protein kinase pathway that is antagonized by epidermal growth factor.** .
          We have demonstrated that interferon alpha2 **[IFNA2]** recombinant IFNalpha at growth inhibitory concentrations enhances the expression and signalling activity of the epidermal growth factor receptor EGF R in human epidermoid carcinoma KB cells.
          Here we report that KB cells exposed to IFNalpha underwent apoptotic cell death and this effect was antagonized by EGF.
          We have also found that IFNalpha enhanced the expression of heat shock proteins HSP HSP 70, HSP 90 and HSP 27 and activated the NH2 terminal Jun kinase 1 JNK 1 and p38 mitogen activated protein kinase, the target enzymes of a stress dependent intracellular transduction pathway.
          Moreover, the overexpression of the wild type JNK 1, obtained through plasmid transfection of KB cells, induced apoptosis which was potentiated by the exposure of wild type JNK 1 JNK 1wt transfected cells to IFNalpha.
          All these effects were neutralized by the addition of EGF to parental and JNK 1wt transfected KB cells exposed to IFNalpha.
          In conclusion, EGF has a protective effect on KB cells from apoptosis while antagonizing a stress response elicited by IFNalpha and targeted on the stress pathway terminal kinases.

          - J Mol Biol, 1999   **Biophysical analysis of the interaction of human ifnar2 expressed in E. coli with IFNalpha2.** .
            Type I interferons are cytokines which activate an anti viral response by binding to two specific cell surface receptors, **ifnar1** and ifnar2.
            Here, we report purification and refolding of the extracellular part of human ifnar2 ifnar2 EC expressed in Escherichia coli and its characterization with respect to its interaction with interferon alpha2 **[IFNA2]** IFNalpha2 .
            The 25 kDa, non glycosylated ifnar2 EC is a stable, fully active protein, which inhibits antiviral activity of IFNalpha2.
            The stoichiometry of binding IFNalpha2 is 1 1, as determined by gel filtration, chemical cross linking and solid phase detection.
            The affinity of this interaction is 10 nM, which is similar to the affinity measured for the cell surface bound ifnar2 receptor.
            No difference in affinity was found throughout various assays using optical detection as BIAcore or reflectometric interference spectorscopy.
            However, the binding kinetics as measured in homogeneous phase by fluorescence de quenching was about three times faster than that measured on a sensor surface.
            The rate of complex formation is relatively high compared to other cytokine receptor interactions.
            The salt dependence of the association kinetics suggest a limited but significant contribution of electrostatic forces towards the rate of complex formation.
            The dissociation constant increases with decreasing pH according to the protonation of a base with a pKa of 6.7.
            The surface properties of the IFNalpha2 binding surface on ifnar2 were interpreted according to the pH and salt dependence of the interaction.
